# Study on Photocuring Printing Technology of Polyurethane Acrylate-Based Transparent Braces

**DOI:** 10.3390/ma19194241

**Published:** 2026-10-07

**Authors:** Fenglian Zhong, Qian Jiang, Liang Guo, Miaoning Yan, Qingmao Zhang

**Affiliations:** 1 School of Optoelectronic Science and Engineering, South China Normal University, Guangzhou 510006, China; 2 Riton Additive Technology Co., Ltd., Guangzhou 510378, China

**Keywords:** 3D printing, light-cured, polyurethane, diluent, transparent brace

## Abstract

A polyurethane acrylate-based photosensitive resin system was developed for 405 nm DLP printing. The formulations were prepared by blending commercial urethane acrylate RT75A with TPO-L, BBOT, and one of four reactive diluents (ACMO, IBOMA, TPGDA, or CTFA). Double-bond conversion was determined by ATR-FTIR, whereas viscosity, UV–Vis transmittance, working-curve parameters (critical exposure and penetration depth), volumetric shrinkage, and mechanical properties were evaluated. The reactive diluent markedly affected both the liquid-state behavior of the resin formulations and the properties of the cured networks. RT75A-TP showed the highest double-bond conversion (97.9%) and volumetric shrinkage (4.65%). RT75A-CT exhibited the highest viscosity at 25 °C and the largest elongation at break (119 ± 9.5%). RT75A-IB exhibited the highest tensile strength (43.3 ± 3.1 MPa) and flexural strength (57.8 ± 1.1 MPa). All formulations maintained transmittance above 80% from 450 to 800 nm. A transparent aligner with a nominal thickness of 0.55 mm was directly printed from RT75A-IB, and its dimensional accuracy was evaluated by 3D scanning. Overall, the results show that reactive-diluent selection enables the coordinated tuning of DLP processability, double-bond conversion, optical performance, and mechanical behavior.

## 1. Introduction

Photocuring 3D printing is a vat-photopolymerization process that enables high-resolution, layer-by-layer manufacture of complex polymer parts and customized production. Among such processes, digital light processing (DLP) cures an entire patterned layer at once and is therefore used in biomedical engineering, aerospace, prototyping, and dental applications [1,2]. During DLP, photoinitiator molecules absorb incident light and generate reactive radicals, which initiate the polymerization of unsaturated double bonds in oligomers and reactive diluent monomers [3]. The material landscape has progressed beyond conventional epoxy acrylates, polyurethane acrylates, and vinyl resins to include biocompatible hydrogel systems [4], functionally graded acrylate networks [5], and conductive photopolymers [6].

Transparent orthodontic aligners are conventionally fabricated by producing a dental master model, thermoforming a polymer film over the model, and then trimming and polishing the appliance [7,8,9]. Direct photocuring additive manufacturing can eliminate the physical master model and reduce manual thermoforming steps, while providing digital control of geometry and nominal thickness. However, it does not remove the need for standardized cleaning, post-curing, fit verification, and material-safety assessment [8,9].

Flexible materials have been used in soft robotics [10], wearable sensors [11], and bioengineering [12]. Nevertheless, a photocurable resin that simultaneously meets the optical, mechanical, processing, and intraoral-use requirements of directly printed transparent aligners has not been fully established. Published studies show that the stiffness, strength, ductility, and stress-relaxation behavior of directly printed resins can differ substantially from those of thermoformed aligner materials, especially after conditioning in a simulated oral environment [12,13]. Thus, transparency or tensile strength alone is not sufficient to establish clinical suitability; dimensional accuracy, retained force, water uptake/swelling, residual-monomer release, and biocompatibility also require assessment before clinical translation.

In the present study, commercial two-component energy-curable urethane acrylate RT75A was used as the oligomeric precursor. TPO-L was used, as the photoinitiator and photoabsorber; the active diluent was the intended variable. ACMO, IBOMA, TPGDA, and CTFA were selected because their molecular structures provide distinct network-forming conditions: ACMO contains carbonyl functionality that may support hydrogen bonding with urethane-containing components; IBOMA introduces a rigid alicyclic group; TPGDA provides two acrylate groups; and CTFA is a monofunctional cyclic acetal acrylate. These structural differences are expected to influence resin viscosity, photopolymerization, network connectivity, chain mobility, and the optical and mechanical response. Their effects should be interpreted together with the experimental data, rather than inferred from degree of conversion alone [14,15].

All formulations use the same RT75A/reactive-diluent/TPO-L mass basis (50/49/1) to isolate the effect of diluent identity rather than to claim a universal optimum ratio. The novelty of this work is the controlled comparison of four chemically distinct active diluents in a common RT75A/TPO-L/BBOT system, linking their structural features to DLP processability (critical exposure and viscosity), double-bond conversion, shrinkage, transparency, and mechanical behavior. The aim is to identify a transparent and printable formulation with a balanced performance profile for subsequent aligner fabrication and to provide a polymer-science basis for formulation optimization.

## 2. Experimental Section

Materials

RT75A (difunctional polyurethane acrylate) purchased from Guangzhou Songda New Materials Co., Ltd., Guangzhou, China. Active diluent monomers include ACMO (Acryloylmorpho—line), IBOMA (Isobornyl methacrylate), TPGDA (Tri(propylene glycol)diacrylate) and CTFA((5-ethyl-1,3-dioxan-5-y1) methyl acrylate), which were purchased from Shanghai Meryl Chemical Co., Ltd. and Shanghai Guangyi Chemical Co., Ltd. (Shanghai, China), respectively. The photoinitiator TPO-L (2,4,6-trimethyl benzoyl phosphonate ethyl ester) and the photoabsorber BBOT (2,5-bis(5-tert-butyl-2-benzoazole) thiophene) were purchased from Shanghai Optoelectronic Chemical Co., Ltd., Guangzhou, China.

**Sample preparation**.

The photosensitive resin formulation was prepared as follows: active diluent monomers—IBOMA, ACMO, TPGDA, and CTFA—were sequentially added to a light-resistant glass bottle. Photoinitiator TPO-L was then incorporated into the mixture, which was homogenized using a mechanical agitator (Shanghai Sunne Co., Ltd., Guangzhou, China). Subsequently, the resin solution was subjected to ultrasonication to remove entrapped air bubbles. All prepared resins were stored in amber glass containers under dark conditions to prevent premature photopolymerization. Table 1 summarizes their respective mass proportions in the formulations.

The prepared photosensitive resin was carefully dispensed into the resin vat of the DLP-based 3D printer (Riton-D190, Guangzhou Biotechnology Co., Ltd., Guangzhou, China). Prior to printing, the critical exposure dose (E_c_) was experimentally determined. To establish a power–cure depth relationship, monolayers were photopolymerized at systematically varied irradiance levels, and the resulting cured layer thicknesses were measured.

3D printing.

The DLP 3D printer employed in this study utilized a 405 nm UV light source for photopolymerization. The STL model was sliced using dedicated software to generate layers of uniform thickness (50 μm). Layer-specific exposure parameters—including irradiance and exposure duration—were determined based on the experimentally measured critical exposure dose (E_c_) and penetration depth (D_p_). Post-processing consisted of the following steps: (i) ultrasonic cleaning in anhydrous ethanol for 5 min; (ii) removal of residual uncured resin via gentle air drying or soft brushing; (iii) thermal drying in a convection oven at ambient temperature; and (iv) secondary post-curing in a 405 nm LED irradiation chamber filled with pure water for 15 min. This protocol yielded specimens with smooth, residue-free surfaces.

Characterization.

The cure depth–exposure-dose relationship was fitted with the Jacobs working curve, derived from Beer–Lambert attenuation [16,17,18]:

(1) $$C_{d}=D_{p}\ln(\frac{E}{E_{c}})$$

Here, Cd is the measured cured-layer thickness (mm); Dp is the penetration depth (mm) obtained from the slope of the linear Cd-versus-ln(E) fit; E is the incident radiant exposure (mJ cm^−2^, irradiance × exposure time); and Ec is the critical radiant exposure (mJ cm^−2^), obtained by extrapolating the fit to Cd = 0. Ec therefore denotes the operational exposure threshold for a measurable cured layer under the 405 nm test conditions, not a direct measure of double-bond conversion, crosslink density, or final mechanical performance. Under a fixed wavelength and formulation, a smaller Dp indicates stronger effective attenuation. These working-curve parameters were interpreted together with the ATR-FTIR conversion data [16,17,18].

**Figure 1:**
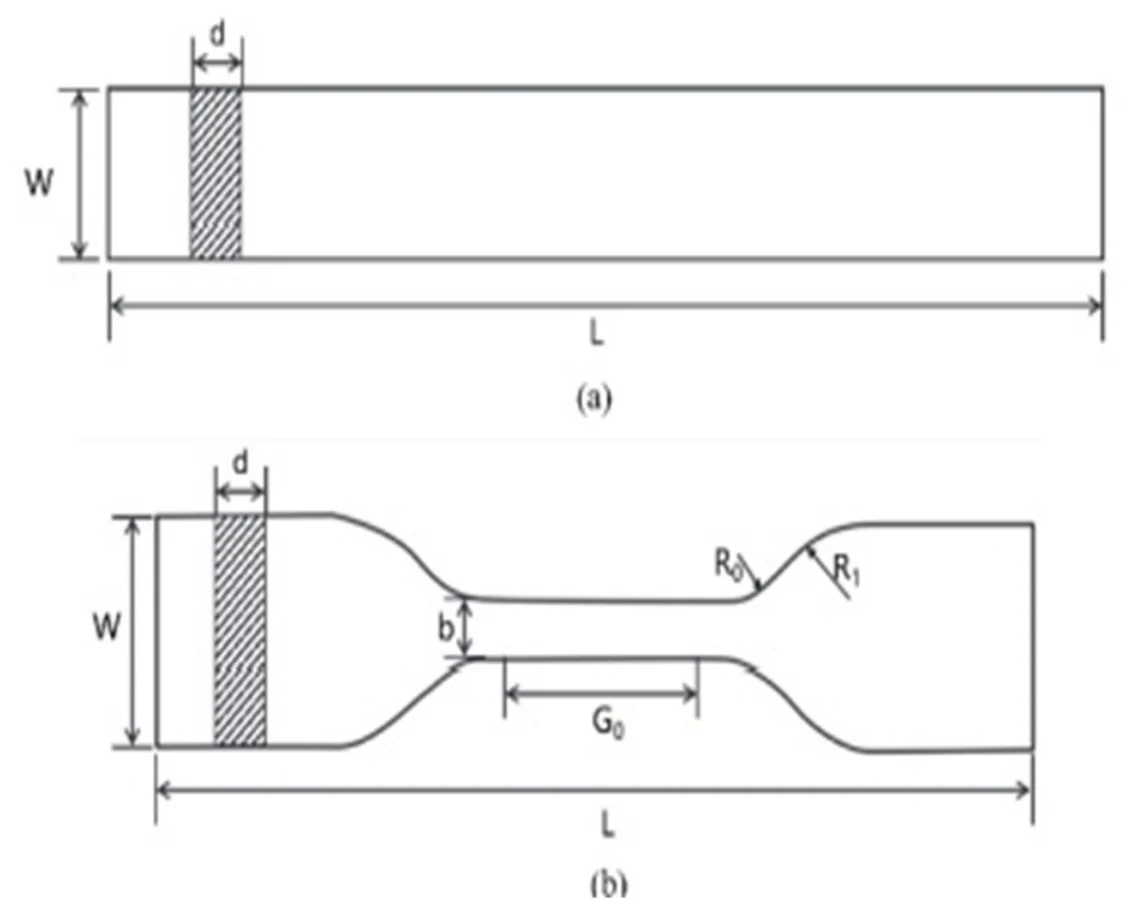
Specimen geometries used for (**a**) flexural and (**b**) tensile tests. L, overall length; W, overall width; d, thickness; b, width of the narrow section; G_0_, gauge length; and R_1_ and R_2_, transition radii.

Tensile and flexural properties were evaluated using a microcomputer-controlled electronic universal testing machine (WDW-1, Shandong Kake Testing Technology Co., Ltd., Jinan, China) at ambient temperature (23 ± 2 °C) and a crosshead speed of 2 mm/min. Five replicate specimens were fabricated and tested per formulation; reported values represent the arithmetic mean ± standard deviation. The specimen geometry is shown in Figure 1, and the corresponding dimensions were designed in accordance with the relevant ASTM standard.

In order to measure the effect of temperature change on viscosity, a Haake rheometer was used to test the rheological properties. Stainless steel parallel plates (25 mm in diameter) with a 1 mm gap were used, the test temperature was 20–70 °C, and the heating rate was 5 °C/min.

The degree of double-bond conversion (DC) was determined by ATR-FTIR spectroscopy from the decrease in the aliphatic C=C stretching band at 1636 cm^−1^, using the intrinsic urethane C=O stretching band at 1706–1713 cm^−1^ as the internal reference. Spectra were collected before and after photocuring, and the peak areas were integrated using identical baseline and wavenumber windows for each formulation. DC was calculated from the normalized C=C/C=O area ratio according to Equation (2):

(2) $$\begin{array}{c} \mathrm{DC}\;=\;1\;-\frac{{A^{\prime }}_{c=c}/{A^{\prime }}_{c=o}}{A_{c=c}/A_{c=o}} \end{array}$$

where A represents the integrated peak area. This normalization expresses the fraction of the initial C=C signal consumed within each formulation and accounts for differences in the initial double-bond content of ACMO, IBOMA, TPGDA, and CTFA [19,20].

And then the dynamics of UV coating system under different irradiation modes were investigated.

The volume shrinkage rate was measured by densitometer (ZMD-2, Shanghai Fangrui Instrument Co., Ltd., Shanghai, China), which measured the density of the liquid before curing and the density of the sample after curing. The formula for calculating volume shrinkage is as follows:

(3) $$\mathrm{volume}\;\mathrm{shrinkage}(\%)\;=\;\frac{\rho_{2}-\rho_{1}}{\rho_{2}}\times 100\%$$

where *ρ*_1_ is the density of the uncured liquid resin before photopolymerization and *ρ*_2_ is the density of the cured specimen after photopolymerization. These definitions clarify the physical meaning and measurement state represented by each term.

## 3. Result and Discussion

Curing kinetics and shrinkage.

**Figure 2:**
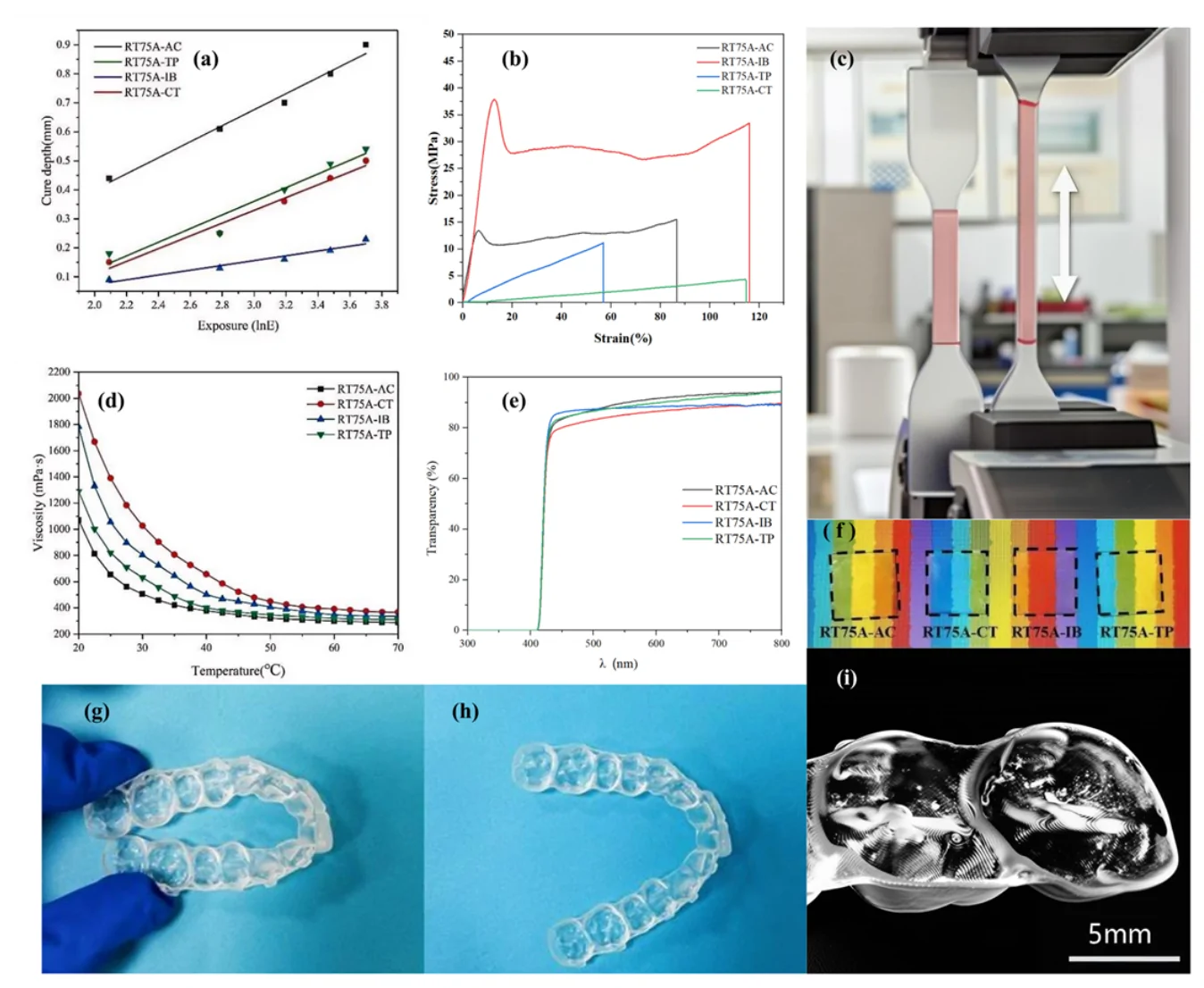
(**a**) Linear fit of critical exposure to cure depth. (**b**) Tensile stress–strain curves of four groups of resin splines. (**c**) Comparison of the specimen before and after the tensile test. (**d**) Viscosity-temperature curve of photosensitive resin with different resin. (**e**) UV–visible light transmission spectrum. (**f**) A digital photo of the prepared transparent elastomer film on the liquid crystal screen. (**g**,**h**) Clear braces can be bent with finger pressing. (**i**) A partial image of direct 3D printing transparent braces.

Figure 2a presents the linear regression of cured depth versus logarithmic exposure dose, as described by Equation (1). The solid line corresponds to the best-fit linear model; its slope represents the penetration depth (D_p_); and its *x*-axis intercept yields the critical exposure dose (E_c_).

The experimentally determined D_p_ and E_c_ values for each resin formulation are summarized in Table 2. Using RT75A as the base oligomer, the incorporation of different active diluents—IBOMA, ACMO, TPGDA, and CTFA—systematically modulates both E_c_ and D_p_. Specifically, the RT75A–ACMO formulation exhibits the lowest E_c_, indicating superior photosensitivity under identical irradiation conditions. In contrast, the RT75A–IBOMA system displays the smallest D_p_, attributable to the pronounced steric hindrance imposed by the isobornyl ester moiety in IBOMA. This structural constraint impedes molecular mobility during photopolymerization, thereby reducing the efficiency of vinyl C=C bond conversion and necessitating higher incident energy to achieve equivalent network formation.

Spectrum analysis.

**Figure 3:**
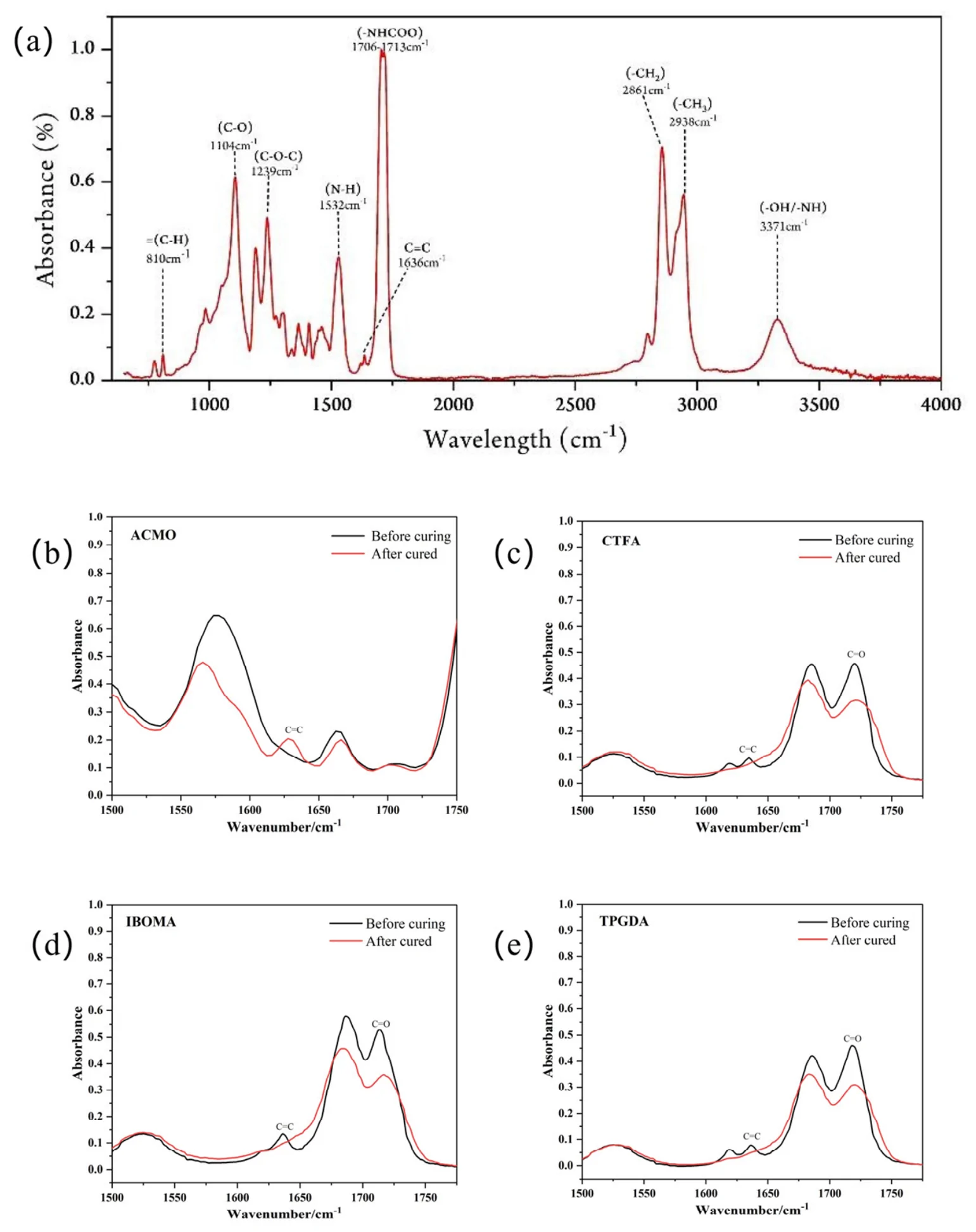
(**a**) ATR-FTIR spectrum of RT75A. Positions of the C=C double-bond and C=O bond peaks before and after curing: (**b**) RT75A-AC, (**c**) RT75A-CT, (**d**) RT75A-IB, and (**e**) RT75A-TP.

The ATR-FTIR spectra can be divided into the functional-group region (4000–1300 cm^−1^) and the fingerprint region (1300–400 cm^−1^). Figure 3a shows the ATR-FTIR spectrum of RT75A. The intrinsic urethane C=O stretching band at 1706–1713 cm^−1^ was used as the internal reference, while the aliphatic C=C stretching band at 1636 cm^−1^ was used to monitor photocuring. The decrease in the normalized C=C/C=O peak-area ratio after irradiation confirms consumption of the polymerizable double bonds [20,21]. The spectra of the four formulations before and after curing are shown in Figure 3b–e.

As summarized in Table 3, RT75A-TP showed the highest double-bond conversion (97.9%) and volumetric shrinkage (4.65%). TPGDA contains two acrylate groups per molecule; thus, its higher functionality provides more polymerizable sites and increases the density of potential network junctions during curing. The resulting high conversion is therefore accompanied by greater volumetric shrinkage. RT75A-AC and RT75A-IB showed comparable conversion values of 83.4% and 82.4%, respectively, whereas RT75A-CT showed the lowest conversion (79.7%) and shrinkage (3.04%). This trend indicates that conversion is governed by the combined effects of functionality, resin viscosity, molecular mobility, and the evolving polymer network, rather than by steric effects alone. In particular, the higher viscosity of the CTFA-containing formulation restricts molecular diffusion during photocuring and contributes to its lower conversion.

Mechanical property characterization.

Figure 2b presents the tensile stress–strain responses of the four photosensitive resins. RT75A-IB exhibited the highest tensile strength (43.3 ± 3.1 MPa) and flexural strength (57.8 ± 1.1 MPa), together with an elongation at break of 105 ± 15.4%. RT75A-AC showed intermediate tensile strength (15.2 ± 2.3 MPa) and elongation at break (83 ± 19.3%). In contrast, RT75A-TP exhibited lower tensile and flexural strengths of 12.0 ± 1.0 MPa and 7.4 ± 0.9 MPa, respectively, with an elongation at break of 54 ± 8.3%. RT75A-CT showed the highest elongation at break (119 ± 9.5%) but the lowest tensile strength (5.2 ± 0.7 MPa), indicating a more compliant network. RT75A-IB and RT75A-AC showed identifiable yield points at approximately 13% and 7% strain, respectively, followed by localized necking and plastic deformation. This behavior indicates that, after yielding, polymer-chain segments undergo orientation and rearrangement under tensile loading. By comparison, RT75A-TP and RT75A-CT showed predominantly elastic deformation before fracture.

The mechanical differences can be interpreted from the structures of the reactive diluents. The rigid isobornyl group in IBOMA restricts local segmental motion and supports a stronger load-bearing network, which is consistent with the highest tensile and flexural strengths of RT75A-IB. ACMO contains a polar morpholine-derived amide structure that can strengthen intermolecular interactions with the urethane-acrylate network, thereby providing a balanced combination of strength and ductility. Although RT75A-TP showed the highest double-bond conversion (97.9%), conversion alone does not determine mechanical strength. The difunctional TPGDA promotes rapid network formation and greater shrinkage; rapid multifunctional polymerization can also generate locally dense crosslinked regions that limit effective chain extension under loading [21,22]. This interpretation is consistent with the lower tensile and flexural strengths and the shorter elongation at break of RT75A-TP. By contrast, the monofunctional CTFA-containing formulation retained the highest elongation at break but the lowest tensile strength, consistent with lower network connectivity.

To compare the energy-absorption capacity of the formulations, tensile toughness (U_t_) was calculated as the area under the engineering stress–strain curve, as shown in Table 4.

(4) $$U_{T}=\int_{0}^{\varepsilon_{f}}\sigma \left(\varepsilon \right)\mathrm{d\varepsilon}$$

where U_t_ is the tensile toughness, σ(ε) is the engineering stress as a function of engineering strain ε, and ε_f_ is the engineering strain at fracture. Thus, the upper integration limit ε_f_ represents the strain at which the specimen fractures [14]. Because engineering strain is dimensionless, the integral represents the energy absorbed per unit volume before fracture and is reported in MJ/m^3^. The curve-derived toughness values show the order RT75A-IB > RT75A-AC > RT75A-TP > RT75A-CT. RT75A-IB therefore combines high strength with sustained deformation, whereas the lower values of RT75A-TP and RT75A-CT reflect their lower stress-bearing capacity over the tested strain range.

In bottom-up DLP printing, resin viscosity governs gap refilling after platform separation and thereby affects recoating time and layer uniformity. Figure 2d shows that the viscosity of all formulations decreases with temperature. At 25 °C, all four resins exhibit viscosities above 500 mPa·s, while RT75A-CT remains the most viscous formulation throughout the measured temperature range and RT75A-AC the least viscous. This ranking shows that the active diluent changes the liquid-state flow behavior of the RT75A system. The higher viscosity of the CTFA-containing formulation likely reflects a less effective dilution of the RT75A oligomer and stronger molecular packing in the uncured mixture. Its viscosity decreases substantially with increasing temperature. Therefore, the processing behavior of each formulation should be interpreted from the complete viscosity–temperature profile together with the selected recoating interval, rather than from a universal viscosity threshold alone [23,24].

Transparent braces: model printing and accuracy testing.

Transparent braces were fabricated by DLP printing using RT75A-IB, which showed a balanced combination of printability, high optical transmittance, and superior tensile and flexural performance among the investigated formulations. The printed aligners remained transparent and retained their structural integrity during manual bending (Figure 2g,h). UV–visible spectroscopy showed that all formulations exhibited transmittance above 80% in the 450–800 nm range (Figure 2e), confirming the optical suitability of RT75A-IB for direct aligner printing.

The 3D scan results showed region-dependent thickness variation in the printed aligner (Table 5). With a designed thickness of 0.55 mm, the mean thicknesses at the lateral, internal, and underside regions were 0.528 ± 0.031, 0.568 ± 0.034, and 0.734 ± 0.052 mm, corresponding to relative deviations of −4.05%, +3.31%, and +33.39%, respectively. The larger thickness at the underside region is consistent with geometry-dependent resin retention and cumulative light exposure in downward-facing regions during layer-by-layer DLP fabrication. Similar site-dependent thickness deviations have been reported for directly printed clear aligners [25,26]. Figure 2i presents the layer-wise surface morphology of the directly printed aligner.

Study Scope and Future Perspectives.

The present study establishes formulation–processing–property relationships for an RT75A-based photocurable resin under controlled conditions. Building on these findings, thermal, dynamic-mechanical, and network characterization could clarify how monomer structure and network evolution govern temperature-dependent behavior. For direct clear-aligner applications, simulated oral aging could reveal changes in dimensional stability, stress relaxation, force retention, fatigue resistance, and optical properties. Chemical and biological analyses could further establish the relationships among conversion, post-curing, residual monomer release, and biocompatibility. Together with comparisons using commercial aligner materials, these studies would define the practical performance range of the resin and guide further optimization of its formulation, printing parameters, and post-curing conditions.

## 4. Conclusions

In conclusion, RT75A-based photosensitive resins containing ACMO, IBOMA, TPGDA, or CTFA were evaluated for 405 nm DLP printing. The reactive diluent regulated the curing, viscosity, optical, and mechanical properties of the formulations. All formulations retained transmittance above 80% between 450 and 800 nm. RT75A-TP showed the highest double-bond conversion (97.9%) and volumetric shrinkage (4.65%), whereas RT75A-CT exhibited the highest viscosity at 25 °C and the largest elongation at break (119.05%). RT75A-IB showed the highest tensile and flexural strengths (43.33 and 57.28 MPa, respectively) and was therefore selected for direct aligner printing. The 0.55 mm RT75A-IB aligner showed region-dependent thickness deviations of −4.05%, +3.31%, and +33.39% at the lateral, internal, and underside regions, respectively. These results demonstrate that reactive-diluent selection enables the coordinated optimization of DLP processability, transparency, and mechanical performance for the direct fabrication of transparent aligners.

## Figures and Tables

**Table 1:** The formula of the photosensitive resin.

Item	Mass Ratio					
	RT75A	ACMO	IBOMA	TPGDA	CTFA	TPO-L
RT75A-AC	50	49	0	0	0	1
RT75A-IB	50	0	49	0	0	1
RT75A-TP	50	0	0	49	0	1
RT75A-CT	50	0	0	0	49	1

**Table 2:** Critical exposure and curing depth from linear fit.

	Item	RT75A-AC	RT75A-CT	RT75A-IB	RT75A-TP
Critical exposure	mJ/cm^2^	1.73	4.50	3.04	4.34
Cure depth	D_p_ (mm)	0.28	0.22	0.08	0.24

**Table 3:** The shrinkage and double bond conversion of the items.

Material	Shrinkage (%)	Double Bond Conversion (%)
RT75A-AC	3.92	83.4
RT75A-CT	3.04	79.7
RT75A-IB	3.32	82.4
RT75A-TP	4.65	97.9

**Table 4:** Mechanical properties.

Item	Tensile Strength (MPa)	Bending Strength (MPa)	Tensile Strain (%)	Shore Hardness (HD)	Tensile Toughness (MJ/m^3^)
RT75A-AC	15.2 ± 2.3	24.8 ± 3.3	83 ± 19.3	70	9.6
RT75A-IB	43.3 ± 3.1	57.8 ± 1.1	105 ± 15.4	68	29.0
RT75A-TP	12.0 ± 1.0	7.4 ± 0.9	54 ± 8.3	56	3.0
RT75A-CT	5.2 ± 0.7	-	119 ± 9.5	25	2.4

**Table 5:** Thickness testing of light-cured transparent braces.

Tooth Number	Lateral Thickness (mm)	Lateral Thickness Standard Deviation	Internal Thickness (mm)	Medial Thickness Standard Deviation	Underside Thickness (mm)	Underside Thickness Average Deviation
3	0.5	0.08	0.59	0.16	0.75	0.06
4	0.51	0.14	0.61	0.22	0.81	0.08
7	0.55	0.19	0.585	0.09	0.7	0.02
8	0.515	0.13	0.58	0.1	0.835	0.13
11	0.5	0.12	0.615	0.11	0.67	0.16
12	0.505	0.13	0.59	0.12	0.7	0.02
15	0.5	0.16	0.525	0.05	0.73	0.12
16	0.58	0.12	0.525	0.03	0.765	0.05
19	0.515	0.03	0.535	0.09	0.68	0.1
20	0.565	0.09	0.535	0.05	0.72	0.14
23	0.565	0.09	0.56	0.06	0.71	0.1

## Data Availability

The original contributions presented in this study are included in the article. Further inquiries can be directed to the corresponding author.

## References

[1] Ligon S.C., Liska R., Stampfl J., Gurr M., Mülhaupt R. (2017). Polymers for 3D Printing and Customized Additive Manufacturing. Chem. Rev..

[2] Zhu G., Hou Y., Xu J., Zhao N. (2021). Reprintable Polymers for Digital Light Processing 3D Printing. Adv. Funct. Mater..

[3] Wu J., Zhao Z., Hamel C.M., Mu X., Kuang X., Guo Z., Qi H.J. (2018). Evolution of material properties during free radical photopolymerization. J. Mech. Phys. Solids.

[4] Grigoryan B., Paulsen S.J., Corbett D.C., Sazer D.W., Fortin C.L., Zaita A.J., Greenfield P.T., Calafat N.J., Gounley J.P., Ta A.H. (2019). Multivascular networks and functional intravascular topologies within biocompatible hydrogels. Science.

[5] Kuang X., Wu J., Chen K., Zhao Z., Ding Z., Hu F., Fang D., Qi H.J. (2019). Grayscale digital light processing 3D printing for highly functionally graded materials. Sci. Adv..

[6] Tsai S.-C., Chen L.-H., Chu C.-P., Chao W.-C., Liao Y.-C. (2022). Photo curable resin for 3D printed conductive structures. Addit. Manuf..

[7] Jindal P., Juneja M., Siena F.L., Bajaj D., Breedon P. (2019). Mechanical and geometric properties of thermoformed and 3D printed clear dental aligners. Am. J. Orthod. Dentofac. Orthop..

[8] Tartaglia G.M., Mapelli A., Maspero C., Santaniello T., Serafin M., Farronato M., Caprioglio A. (2021). Direct 3D Printing of Clear Orthodontic Aligners: Current State and Future Possibilities. Materials.

[9] Yu X., Li G., Zheng Y., Gao J., Fu Y., Wang Q., Huang L., Pan X., Ding J. (2022). ‘Invisible’ orthodontics by polymeric ‘clear’ aligners molded on 3D-printed personalized dental models. Regen. Biomater..

[10] Ghosh K., Kar-Narayan S. (2024). 3D-printed photothermal-responsive shape-memory polymer for soft robotic applications. Chem. Commun..

[11] Peng S., Thirunavukkarasu N., Chen J., Zheng X., Long C., Huang X., Weng Z., Zheng L., Wang H., Peng X. (2023). Vat photopolymerization 3D printing of transparent, mechanically robust, and self-healing polyurethane elastomers for tailored wearable sensors. Chem. Eng. J..

[12] Lee S.Y., Kim H., Kim H.J., Chung C.J., Choi Y.J., Kim S.J., Cha J.Y. (2022). Thermo-mechanical properties of 3D printed photocurable shape memory resin for clear aligners. Sci. Rep..

[13] Shirey N., Mendonca G., Groth C., Kim-Berman H. (2023). Comparison of mechanical properties of 3-dimensional printed and thermoformed orthodontic aligners. Am. J. Orthod. Dentofac. Orthop..

[14] Huang W., Zhang J., Singh V., Xu L., Kabi P., Bele E., Tiwari M.K. (2023). Digital light 3D printing of a polymer composite featuring robustness, self-healing, recyclability and tailorable mechanical properties. Addit. Manuf..

[15] Lublin D., Hao T., Malyala R., Kisailus D. (2024). Multiscale mechanical characterization of biobased photopolymers towards sustainable vat polymerization 3D printing. RSC Adv..

[16] Li Y., Mao Q., Yin J., Wang Y., Fu J., Huang Y. (2021). Theoretical prediction and experimental validation of the digital light processing (DLP) working curve for photocurable materials. Addit. Manuf..

[17] Hofstetter C., Orman S., Baudis S., Stampfl J. (2018). Combining cure depth and cure degree, a new way to fully characterize novel photopolymers. Addit. Manuf..

[18] Lipkowitz G., Saccone M.A., Panzer M.A., Coates I.A., Hsiao K., Ilyn D., Kronenfeld J.M., Tumbleston J.R., Shaqfeh E.S.G., DeSimone J.M. (2024). Growing three-dimensional objects with light. Proc. Natl. Acad. Sci. USA.

[19] Delgado A.H.S., Young A.M. (2021). Methacrylate peak determination and selection recommendations using ATR-FTIR to investigate polymerisation of dental methacrylate mixtures. PLoS ONE.

[20] Manoukakis T., Nikolaidis A.K., Koulaouzidou E.A. (2024). Polymerization kinetics of 3D-printed orthodontic aligners under different UV post-curing conditions. Prog. Orthod..

[21] Choi W.-C., Gavande V., Kim D.-Y., Lee W.-K. (2023). Study on Press Formability and Properties of UV-Curable Polyurethane Acrylate Coatings with Different Reactive Diluents. Polymers.

[22] Gasper S.M., Schissel D.N., Baker L.S., Smith D.L., Youngman R.E., Wu L.-M., Sonner S.M., Hancock R.R., Hogue C.L., Givens S.R. (2006). Integrated Approach to Studying the Development and Final Network Properties of Urethane Acrylate Coatings. Macromolecules.

[23] Choi J.W., Kim G.-J., Hong S., An J.H., Kim B.-J., Ha C.W. (2022). Sequential process optimization for a digital light processing system to minimize trial and error. Sci. Rep..

[24] Šimunović L., Brenko L., Marić A.J., Meštrović S., Haramina T. (2025). Rheology of Dental Photopolymers for SLA/DLP/MSLA 3D Printing. Polymers.

[25] Edelmann A., English J.D., Chen S.J., Kasper F.K. (2020). Analysis of the thickness of 3-dimensional-printed orthodontic aligners. Am. J. Orthod. Dentofac. Orthop..

[26] Bandić R., Vodanović K., Kekez I.V., Mikić I.M., Galić I., Govorko D.K. (2024). Thickness Variations of Thermoformed and 3D-Printed Clear Aligners. Acta Stomatol. Croat. Int. J. Oral Sci. Dent. Med..

